# **Matrix-assisted laser desorption/ionization mass spectrometry imaging for quorum sensing**

**DOI:** 10.1186/s13568-024-01703-6

**Published:** 2024-04-25

**Authors:** Christel Kuik, Sanne W.G. van Hoogstraten, Jacobus J.C. Arts, Maarten Honing, Berta Cillero-Pastor

**Affiliations:** 1 Maastricht MultiModal Molecular Imaging institute (M4i), Maastricht University, Maastricht, the Netherlands; 2 Department of Orthopaedic Surgery, Laboratory for Experimental Orthopaedics, CAPHRI, Maastricht University Medical Centre, Maastricht, the Netherlands; 3 Department of Biomedical Engineering, Orthopaedic Biomechanics, Eindhoven University of Technology, Eindhoven, the Netherlands; 4 Department of Cell Biology-Inspired Tissue Engineering, MERLN Institute for Technology-Inspired Regenerative Medicine, Maastricht University, Maastricht, The Netherlands

**Keywords:** Biofilm, Quorum sensing, Matrix-assisted laser desorption ionization mass spectrometry imaging

## Abstract

Quorum sensing (QS) is a complex communication system in bacteria, directing their response to the environment. QS is also one of the main regulators of bacterial biofilms’ formation, maturation and dispersion. Matrix-assisted laser desorption ionization (MALDI) mass spectrometry imaging (MSI) is a molecular imaging technique that allows the mapping of QS molecules in bacterial biofilms. Here, we highlight the latest advances in MALDI-MSI in recent years and how this technology can improve QS understanding at the molecular level.

## Bacterial biofilm infections and quorum sensing

Biofilms consist of bacterial communities embedded in an extracellular matrix and occur when bacteria colonize implants or tissue surfaces. The extracellular matrix is composed of water and extracellular polymeric substances (EPS), which mainly consist of eDNA, proteins, lipids, nucleic acids, and polysaccharides. Biofilm-associated infections are challenging to treat with conventional antibiotics as the exopolysaccharides in the EPS protect the bacteria from bactericidal concentrations of antibiotics by acting as a diffusion barrier. (Shah et al. 2019) (Pinto et al. 2020) Quorum sensing (QS) is a complex intra- and inter-bacterial communication system which plays an important role in biofilm formation and dispersion. QS regulates gene expression and metabolic exchange among bacteria, influencing cell density and growth.(Pitchapa et al. 2022) Regulation or inhibition of bacterial quorum sensing, can offer a valuable treatment strategy for biofilms. However, detailed information about complex QS systems and metabolites in different bacterial species is limited, and new QS molecules are continuously being discovered. Therefore, a better insight into the mechanism of bacterial QS pathways is needed, as it can lead to potential strategies for preventing the formation and maturation of biofilms.(Ricciardi et al. 2020).

## Expanding molecular coverage in biofilm imaging

Imaging techniques, such as confocal laser scanning microscopy (CLSM), have been widely used to study bacterial biofilms and QS molecules.(Akbari et al. 2023) However, these techniques usually require labelling. On the contrary, mass spectrometry imaging (MSI) can track the distribution of hundreds of molecules and elements on biological surfaces without the need of labelling.(Dunham et al. 2018).

**Fig. 1:**
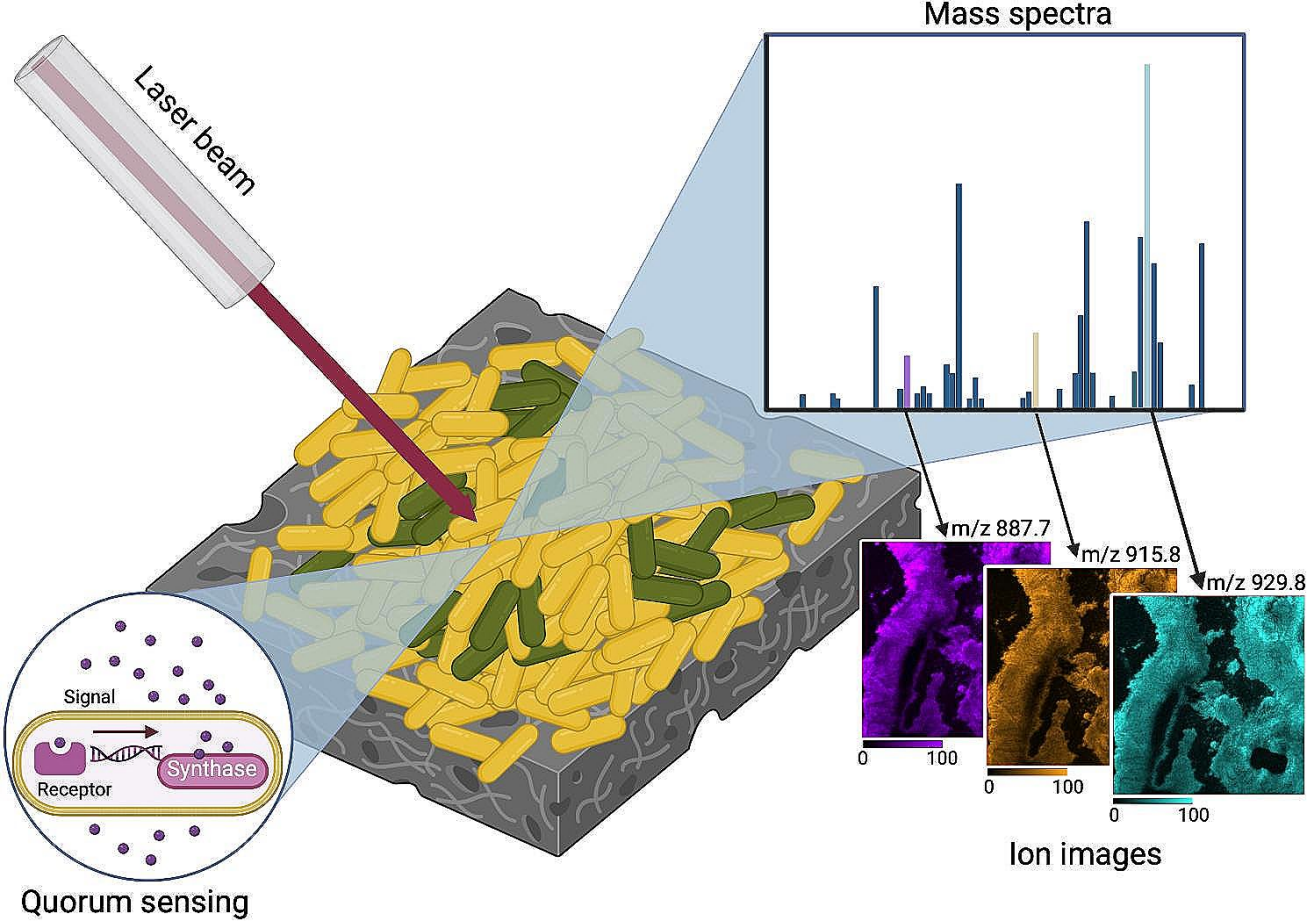
Schematic visualization of a MALDI measurement analyzing QS events in a bacterial biofilm. Middle: A laser impacts the bacterial biofilm surface. The laser ionizes the QS molecules, and the ions are analyzed by the mass spectrometer. Right: The mass spectra of single coordinates are combined into ion images, showing MALDI-MSI images of different lipids in *S. aureus* biofilm grown for four days. These images were generated in positive mode on a Rapiflex (Bruker Daltonics) at 20 μm of lateral resolution using sublimed 2,5-Dihydroxybenzoic acid as a MALDI matrix. Bottom left: a simplified visualization of a QS event. This figure is created with BioRender.com

**Fig. 2:**
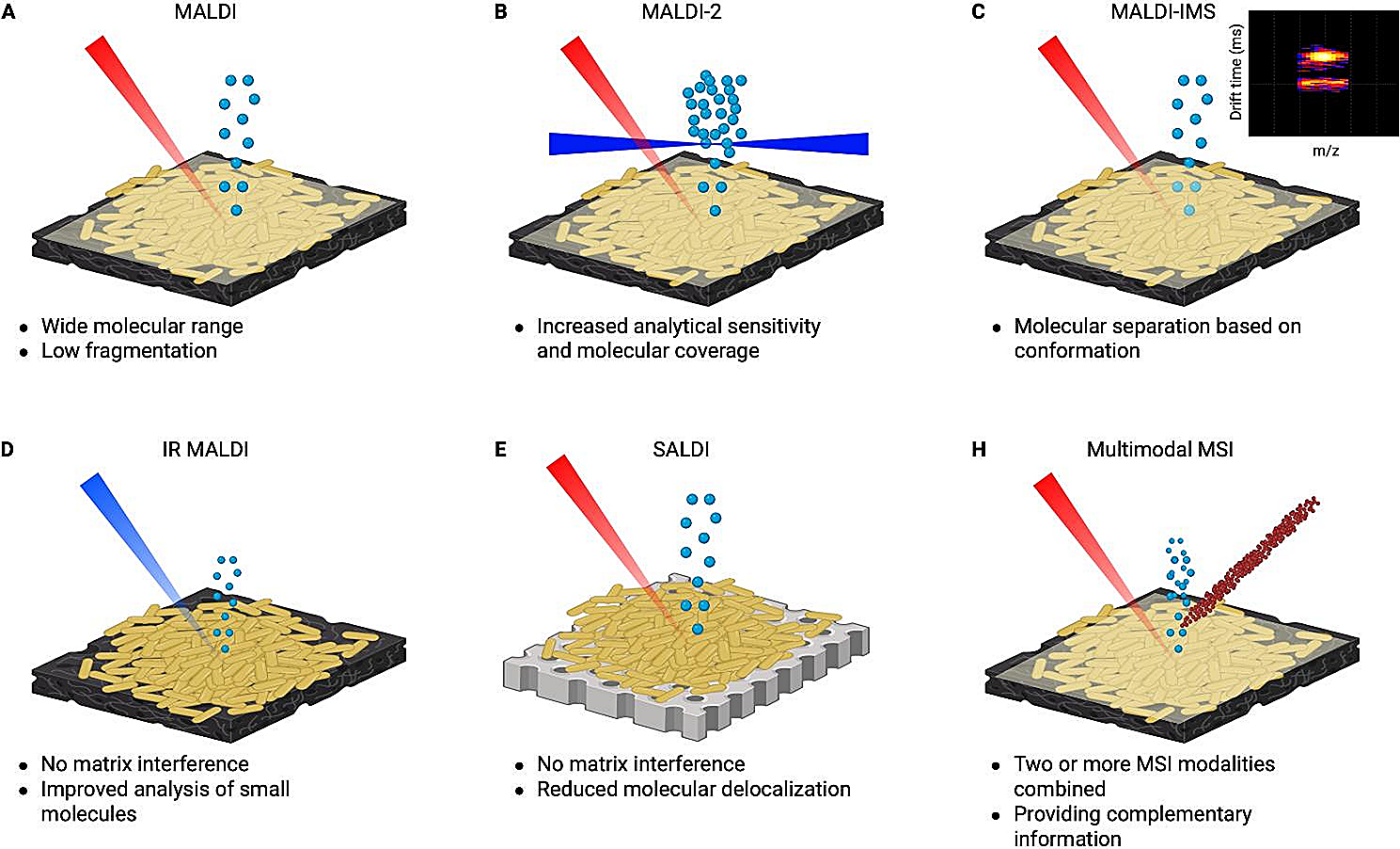
An overview of new advantages in MSI analysis to visualize QS molecules in bacterial biofilms. All techniques are envisioned in a simplified and schematic manner. (**A**) Schematic visualization of classical MALDI-MSI. (**B**) Schematic visualization of MALDI-2. The ion cloud, created by the MALDI laser, will be impacted by a second laser. (**C**) Visualization of MALDI-IMS including an example of an IMS heat map visualizing the separation of molecules with a similar m/z based on their drift time. The ion mobility cell adds an extra separation to the ions created by MALDI. (**D**) A schematic visualization of IR-MALDI. An IR laser impacts the surface and eliminates the need for MALDI matrices. (**E**) A schematic visualization of SALDI. The analytes are transferred from the sample substrate to an assisting membrane. (**F**) Schematic visualization of a multimodal MSI approach using SIMS and MALDI, respectively. Part of this figure is created with BioRender.com

Matrix-assisted laser desorption ionization (MALDI) is the most widely MSI method used in the biomedical field. In MALDI, a laser impacts the surface of a sample where molecules are desorbed and ionized (Figs. 1, 2A). MALDI has already been applied for multiple microbiological applications, including bacterial imaging, providing novel insights into QS signalling, distribution, and metabolite quantification.(Feucherolles and Frache 2022; McCaughey et al. 2023) Indeed, MALDI has been shown as a valuable technique to gain insight into QS pathways within biofilms, as it, for example, revealed the existence of an N-Acyl-homoserine lactone (AHL) QS system in *Pseudomonas putida* biofilms, coordinating bacterial cell density during biofilm maturation, and a relationship between biofilm development and spatial production of QS metabolites has been shown.(Pitchapa et al. 2022) The AHL signalling involved in QS of gram-negative bacteria has been extensively studied due to the involvement in physiological activities of the biofilm, such as nutrient acquisition, conjugation and biofilm formation. However, with standard MALDI-MSI only a fraction of the bacterial metabolome is detected as many QS molecules bear only minor mass differences, due to matrix interference or low technical sensitivity. For instance, the analysis of 2-alkyl-4-quinolones (AQ), can be challenging with MALDI-MSI. AQs are vital QS metabolites for gram-negative bacteria, mainly *Pseudomonas aeruginosa*, as they regulate gene expression for virulence factors, giving competitive advantage to the bacteria producing the AQs. However, this hurdle has been overcome by the development of laser post-ionization (MALDI-2) technology. In this method, a second laser intersects the ion cloud created by the MALDI laser ionizing neutrals (Fig. 2B), allowing the detection of low abundant AQs.(Brockmann et al. 2019) AQ molecules can induce cytotoxicity to host cells, and with MALDI-2, it has been observed that AQ-signals are strongly upregulated in the immediate contact zone, playing a crucial role in bacterial surface attachment and host surface colonization. In addition, MALDI-2 has been used to visualize AQ exchange between *Staphylococcus. aureus* and *P. aeruginosa*.(Brockmann et al. 2019) Gaining insight into the metabolic interaction between competing microbial species can help develop antimicrobial strategies, as co-infections of two pathogens can reduce antimicrobial therapy options.(Scoffone et al. 2019) Furthermore, the challenge of AQ identification can be now resolved by utilizing ion mobility separation (IMS) hyphenated with MALDI-IMS, as depicted in Fig. 2C. IMS separates molecules based on their conformation in the gas phase improving specificity in molecular identification.(Rivera et al. 2022) Recently, trapped ion mobility spectrometry (TIMS) combined with MALDI-2 has been used in bacterial biofilm analysis, leading to a threefold increase in lipid detection.(Rivera et al. 2022) This method shows great potential in analyzing a wider range of QS molecules and metabolites that will improve the understanding of bacterial communication. Another approach to enhance the detection of nucleobases and AQs, is the utilization of infrared (IR) IR-MALDI (see Fig. 2D). IR-MALDI offers the analysis of a different set of molecules than conventional MALDI. IR light is absorbed by O-H and N-H stretching vibrations, enabling water to serve as an endogenous MALDI matrix.(Brockmann et al. 2021) Additionally, the lower absorption coefficient of IR-MALDI permits a deeper penetration into the sample, thereby allowing the analysis of cytosolic compounds in Gram-positive bacteria. Recently, IR-MALDI was compared to traditional MALDI to analyze *P. aeruginosa* and *S. aureus* biofilms. The results showed a reduced background and enhanced detection of AQ molecules. Furthermore, IR-MALDI has been used to image the metabolic response of *P. aeruginosa* to antibiotics. (Brockmann et al. 2021) However, some QS molecules contain a highly conjugated system, such as ring-based molecules, and can directly absorb the UV light of the laser. For example, indole (aromatic, heterocyclic, organic compound) is produced by bacteria and can inhibit QS and virulence factor production. Such molecules are ideal candidates for laser desorption ionization (LDI), where conjugated molecules can be analyzed directly without matrix application.

Most studies using MALDI-MSI for QS detection have predominantly focused on gram-negative bacteria due to their use of small signalling molecules as autoinducers. However, it is noteworthy that gram-positive bacteria employ auto-inducing peptides (AIP), which have not yet been analyzed in biofilms by MALDI(MSI). This gap may be due to their low abundance in the biofilm, leading to ionization competition with highly abundant peptides and proteins. Nevertheless, the adoption of advanced MALDI techniques, such as MALD-2, holds promise in enhancing the sensitivity of AIP detection in biofilms.(McMillen et al. 2021).

When imaging QS metabolites in bacterial biofilm, preservation of the biofilm morphology is crucial. Even though MALDI-MSI measurements of biofilms directly from the culture media are reported, this method is not favourable since, for example, the agar-grown colonies need to be transferred to MALDI-compatible slides, introducing potential molecular delocalization. Alternatively, surface-assisted laser desorption/ionization (SALDI) MSI, combined with the imprinting of the sample to transfer the analytes to appropriate support before analysis, can provide an alternative preserving the molecular distribution (Fig. 2E).(Müller et al. 2022) This method showed a significant improvement in sample preparation and has been applied to image metabolite secretion, mainly focused on lipopeptides in co-cultures of *Bacillus* and *Pseudomonas.* (Müller et al. 2022) Another sample preparation challenge is related to sample height, which can result in peak shifting. Different methods have been developed to overcome some of these artefacts. An example is the embedment of agar-grown biofilms in carboxymethylcellulose, followed by cryosectioning.(Rivera et al. 2022) When the biofilm morphology is well preserved, 3D MALDI imaging can be used to investigate new antibiotic treatments, by providing information on the penetration of active compounds into the biofilm while following bacterial response by detecting metabolic changes. However, this method has not yet been applied in biofilm imaging.

When studying QS metabolites in conjunction with other biomolecules like peptides or proteins, multimodal approaches can be employed. These approaches involve the utilization of two or more molecular imaging modalities to gather complementary information on a single sample (Fig. 2H). Complementary data, such as metabolite analysis by MALDI-MSI, can be combined with fluorescence imaging to detect QS metabolites in association with protein abundance.(Si et al. 2016) Recently, a protocol was published combining fluorescence in situ hybridization (FISH) imaging and MALDI-MSI to enable metabolite research in host-microbe interactions down to single-cell resolution.(Bourceau et al. 2023) In another study, the excretion of rhamnolipid in wild-type and quorum sensing-deficient cells has been analyzed in an multimodal approach.(Masyuko et al. 2014) For this, a combination of MALDI and confocal raman microscopy/spectroscopy (CRM) was employed, for respectively rhamnolipid identification and DNA/RNA-related spectral feature detection. Other multimodal approaches combine MALDI-MSI with high spatial resolution secondary ion mass spectrometry (SIMS), providing insight into metabolic changes at different scales. For instance, a MALDI-guided SIMS method has been used to analyze bioactive secondary metabolites in *Pseudomonas aeruginosa*, explicitly focusing on rhamnolipids and quinolones. (Lanni et al. 2014) This approach revealed the chemical heterogeneity at both macroscopic and cellular levels and shed light on the significance of QS in *Pseudomonas aeruginosa*. Finally, an exciting and growing field is the area of mass spectrometry-based metabolomics is the accessible and open data for metabolite annotation, such as https://metaspace2020.eu, allowing the inclusion of spatial information.(Palmer et al. 2017).

In conclusion, new advances in MALDI-MSI, such as MALDI-2, SALDI-MSI, or multimodal imaging, have improved the detection of QS molecules by increasing molecular coverage, sensitivity, and morphological preservation of the biofilm. Gaining more insight into biofilm formation and interplay with QS mechanisms can lead to new potential approaches against biofilm formation.

**Table:** 

Technique	Advantages	Limitations	Reference
MALDI	Wide molecular range. Low molecular fragmentation.	Suffers from matrix interference in low molecular range.	(Calvano et al. 2018)
MALDI-2	Increases analytical sensitivity. Increases molecular coverage.	Increased data complexity.	(Soltwisch et al. 2015)
MALDI-IMS	Provides separation based on molecular conformation. Increases analytical sensitivity.	Increased data complexity.	(Rivera et al. 2022; Sans et al. 2018)
IR-MALDI	No matrix interference. Improved analysis of small molecules.	Less sensitive compared to UV-MALDI.	(Pirkl et al. 2012; Brockmann et al. 2021)
SALDI	No matrix interference. Reduces molecular delocalization.	Uneven sample distribution can lead to ‘dark areas’ in the ion image.	(Müller et al. 2022)
Multimodal MSI	Provides complementary information on the same sample.	Precise correlation of the images is challenging.	(Neumann et al. 2020)

## Abbreviations

AHL: N-Acyl-homoserine lactone
AQ: 2-alkyl-4-quinolones
CLSM: Confocal laser scanning microscopy
CRM: Confocal raman microscopy/spectroscopy
EPS: Extracellular polymeric substances
FISH: Fluorescence in situ hybridization
IMS: Ion mobility separation
IR: Infrared
LDI: Laser desorption ionization
MALDI: Matrix-assisted laser desorption ionization
MALDI-2: Matrix-assisted laser desorption ionization laser post-ionization
MSI: Mass spectrometry imaging
QS: Quorum sensing
SALDI: Surface-assisted laser desorption/ionization
SIMS: Spatial resolution secondary ion mass spectrometry
TIMS: Trapped ion mobility spectrometry

## References

[CR1] Akbari A, Galstyan A, Peterson RE, Arlinghaus HF, Tyler BJ (2023). Label-free sub-micrometer 3D imaging of ciprofloxacin in native-state biofilms with cryo-time-of-flight secondary ion mass spectrometry. Anal Bioanal Chem.

[CR2] Bourceau P, Geier B, Suerdieck V, Bien T, Soltwisch J, Dreisewerd K (2023). Visualization of metabolites and microbes at high spatial resolution using MALDI mass spectrometry imaging and in situ fluorescence labeling. Nat Protoc.

[CR4] Brockmann EU, Steil D, Bauwens A, Soltwisch J, Dreisewerd K (2019). Advanced methods for MALDI-MS imaging of the Chemical Communication in Microbial communities. Anal Chem.

[CR3] Brockmann EU, Potthoff A, Tortorella S, Soltwisch J, Dreisewerd K (2021). Infrared MALDI Mass Spectrometry with Laser-Induced postionization for imaging of bacterial colonies. J Am Soc Mass Spectrom.

[CR5] Calvano CD, Monopoli A, Cataldi TRI, Palmisano F (2018). MALDI matrices for low molecular weight compounds: an endless story?. Anal Bioanal Chem.

[CR6] Dunham SJB, Ellis JF, Baig NF, Morales-Soto N, Cao T, Shrout JD (2018). Quantitative SIMS Imaging of Agar-based Microbial communities. Anal Chem.

[CR7] Feucherolles M, Frache G (2022) MALDI Mass Spectrometry Imaging: a potential game-changer in a modern Microbiology. Cells 11(23). 10.3390/cells11233900 10.3390/cells11233900 PMC9738593 36497158

[CR8] Lanni EJ, Masyuko RN, Driscoll CM, Aerts JT, Shrout JD, Bohn PW (2014). MALDI-guided SIMS: multiscale imaging of metabolites in bacterial biofilms. Anal Chem.

[CR9] Masyuko RN, Lanni EJ, Driscoll CM, Shrout JD, Sweedler JV, Bohn PW (2014). Spatial organization of Pseudomonas aeruginosa biofilms probed by combined matrix-assisted laser desorption ionization mass spectrometry and confocal Raman microscopy. Analyst.

[CR10] McCaughey CS, Trebino MA, McAtamney A, Isenberg R, Mandel MJ, Yildiz FH (2023). A label-free approach for relative spatial quantitation of c-di-GMP in microbial biofilms. bioRxiv.

[CR11] McMillen JC, Gutierrez DB, Judd AM, Spraggins JM, Caprioli RM (2021). Enhancement of Tryptic Peptide Signals from tissue sections using MALDI IMS postionization (MALDI-2). J Am Soc Mass Spectrom.

[CR12] Müller WH, McCann A, Arias AA, Malherbe C, Quinton L, De Pauw E et al (2022) Imaging metabolites in Agar-based Bacterial Co‐Cultures with minimal Sample Preparation using a DIUTHAME membrane in Surface‐assisted laser Desorption/Ionization Mass Spectrometry**. ChemistrySelect 7(18). 10.1002/slct.202200734

[CR13] Neumann EK, Djambazova KV, Caprioli RM, Spraggins JM (2020). Multimodal Imaging Mass Spectrometry: Next Generation Molecular Mapping in Biology and Medicine. J Am Soc Mass Spectrom.

[CR14] Palmer A, Phapale P, Chernyavsky I, Lavigne R, Fay D, Tarasov A (2017). FDR-controlled metabolite annotation for high-resolution imaging mass spectrometry. Nat Methods.

[CR15] Pinto RM, Soares FA, Reis S, Nunes C, Van Dijck P (2020). Innovative strategies toward the Disassembly of the EPS Matrix in Bacterial Biofilms. Front Microbiol.

[CR16] Pirkl A, Soltwisch J, Draude F, Dreisewerd K (2012). Infrared matrix-assisted laser desorption/ionization orthogonal-time-of-flight mass spectrometry employing a cooling stage and water ice as a matrix. Anal Chem.

[CR17] Pitchapa R, Dissook S, Putri SP, Fukusaki E, Shimma S (2022) MALDI Mass Spectrometry Imaging reveals the existence of an N-Acyl-homoserine Lactone Quorum sensing system in Pseudomonas putida Biofilms. Metabolites 12(11). 10.3390/metabo12111148 10.3390/metabo12111148 PMC9697013 36422288

[CR18] Ricciardi BF, Muthukrishnan G, Masters EA, Kaplan N, Daiss JL, Schwarz EM (2020). New developments and future challenges in prevention, diagnosis, and treatment of prosthetic joint infection. J Orthop Res.

[CR19] Rivera ES, Weiss A, Migas LG, Freiberg JA, Djambazova KV, Neumann EK (2022). Imaging mass spectrometry reveals complex lipid distributions across Staphylococcus aureus biofilm layers. J Mass Spectrom Adv Clin Lab.

[CR20] Sans M, Feider CL, Eberlin LS (2018). Advances in mass spectrometry imaging coupled to ion mobility spectrometry for enhanced imaging of biological tissues. Curr Opin Chem Biol.

[CR21] Scoffone VC, Trespidi G, Chiarelli LR, Barbieri G, Buroni S (2019) Quorum sensing as Antivirulence Target in cystic fibrosis pathogens. Int J Mol Sci 20(8). 10.3390/ijms20081838 10.3390/ijms20081838 PMC6515091 31013936

[CR22] Shah S, Gaikwad S, Nagar S, Kulshrestha S, Vaidya V, Nawani N (2019). Biofilm inhibition and anti-quorum sensing activity of phytosynthesized silver nanoparticles against the nosocomial pathogen Pseudomonas aeruginosa. Biofouling.

[CR23] Si T, Li B, Zhang K, Xu Y, Zhao H, Sweedler JV (2016). Characterization of Bacillus subtilis colony Biofilms via Mass spectrometry and fluorescence imaging. J Proteome Res.

[CR24] Soltwisch J, Kettling H, Vens-Cappell S, Wiegelmann M, Müthing J, Dreisewerd K (2015). Mass spectrometry imaging with laser-induced postionization. BIOANALYSIS.

